# Special Issue “Clinical Epidemiology of Diabetes and Its Complications”

**DOI:** 10.3390/jcm11154510

**Published:** 2022-08-02

**Authors:** Tatsuya Fukuda

**Affiliations:** 1 Department of Endocrinology and Metabolism, Tokyo Metropolitan Health and Hospitals Corporation Okubo Hospital, Tokyo 160-8488, Japan; fukumem@tmd.ac.jp; 2 Department of Molecular Endocrinology and Metabolism, Graduate School of Medical and Dental Sciences, Tokyo Medical and Dental University, Tokyo 113-8519, Japan

The purpose of this Special Issue, “***Clinical Epidemiology of Diabetes and Its Complications***” is to bring more attention to diabetes and its complications and share the latest findings with the medical community.

Coronavirus disease 2019 (COVID-19), which is caused by severe acute respiratory syndrome coronavirus 2 (SARS-CoV-2), emerged at the end of 2019, spread rapidly worldwide, and continues to remain a public health crisis as of 2022 [1]. Notably, the COVID-19 pandemic apparently makes glycemic control more difficult in patients with diabetes. A study from India compared 282 patients who were newly diagnosed with diabetes during the COVID-19 pandemic and 273 patients who were newly diagnosed with diabetes before the pandemic [2]; this study found that patients diagnosed with diabetes during the COVID-19 pandemic had higher levels of fasting and postprandial blood glucose and glycated hemoglobin compared with those diagnosed before the pandemic. Additionally, another systematic review elaborated on the possibility of COVID-19 lockdowns worsening glycemic control in patients with type 2 diabetes [3], potentially, in part, due to a reduction in physical activity and limited access to a healthy diet. Furthermore, the pandemic made it difficult for patients with diabetes to regularly visit hospitals due to fear of contracting the virus, thereby hampering their ability to adequately manage not only their blood glucose levels but also the associated complications. For example, it has been reported that regular screening and early treatment for diabetic retinopathy could prevent vision loss [4,5,6,7]; however, the COVID-19 pandemic led to a decrease in hospital visits and treatment, presumably worsening ophthalmologic prognosis [8]. The diagnosis and treatment of diabetic complications, including retinopathy, are highly dependent on the medical equipment in hospitals; therefore, it may be difficult to manage diabetic complications through telemedicine, even though it has gained traction during the COVID-19 epidemic.

At present, diabetes is widely regarded as a risk factor for serious outcomes in COVID-19, especially among those with poor glycemic control, because they are vulnerable to severe COVID-19 [9]. A large-scale study from the United Kingdom reported a 1.8-fold greater risk of death from COVID-19 among individuals with diabetes compared with that among those without diabetes [10], and showed that the presence of diabetic complications, including cardiovascular disease and nephropathy, was independently associated with risk of death due to COVID-19. Similarly, a cohort study in the entire population of Scotland also reported diabetic retinopathy and nephropathy to be independently associated with fatal or critical-care-requiring cases of COVID-19 [11]. Thus, these studies indicate that management of diabetic complications is as important as glycemic control for the prevention of severe COVID-19.

These findings imply that patients with diabetes are at risk of severe sequelae and are thus required to achieve better control of not only their blood glucose levels but also complications, especially in situations that are more difficult to manage than they were before the COVID-19 pandemic. Therefore, physicians have been asked to more carefully manage blood glucose levels and diagnose and treat complications earlier, in a manner that lowers the risk of coronavirus exposure as much as possible. The first article in this issue, by Saunajoki et al. [12], which is a cross-sectional survey of 496 individuals without a history of either prediabetes or diabetes, demonstrated that plasma glucose levels measured 1 h after a 75 g oral glucose tolerance test were apparently the best glycemic predictor of the presence of albuminuria. This finding may suggest that the blood glucose level 1 h after a 75 g oral glucose tolerance test is predictive of greater risk of kidney disease.

The present Special Issue aims to provide a broad updated spectrum of knowledge of the pathogenetic, diagnostic, and therapeutic aspects of diabetes and its complications, and treatment strategies that remain effective in the COVID-19 pandemic.
